# Cinnamaldehyde Supplementation Reverts Endothelial Dysfunction in Rat Models of Diet-Induced Obesity: Role of NF-E2-Related Factor-2

**DOI:** 10.3390/antiox12010082

**Published:** 2022-12-30

**Authors:** Cristina M. Sena, Ana Pereira, Raquel M. Seiça

**Affiliations:** Institute of Physiology, iCBR, Faculty of Medicine, University of Coimbra, Sub-unidade 1, Pólo III, Azinhaga de Santa Comba, Celas, 3000-548 Coimbra, Portugal

**Keywords:** endothelial dysfunction, obesity, inflammation, cinnamaldehyde, Nrf2 activation, metabolic syndrome

## Abstract

Cinnamaldehyde (CN) is an activator of NF-E2-related factor 2 (Nrf2), which has the potential to reduce endothelial dysfunction, oxidative stress and inflammation in metabolic disorders. Our main purpose was to evaluate the effects of CN on vascular dysfunction in metabolic syndrome rats. Normal Wistar (W) rats were divided into eight groups: (1) Wistar (W) rats; (2) W rats fed with a high-fat diet (WHFD); (3) W rats fed with a sucrose diet (WS); (4) WHFD fed with a sucrose diet (WHFDS); (5) W treated with CN (WCn); (6) WS treated with CN (WSCn); (7) WHFD treated with CN (WHFDCn); (8) WHFDS treated with CN (WHFDSCn). CN treatment with 20 mg/kg/day was administered for 8 weeks. Evaluation of metabolic profile, inflammation, endothelial function, oxidative stress, eNOS expression levels and Nrf2 activation was performed. The metabolic dysfunction was greatly exacerbated in the WHFDS rats, accompanied by significantly higher levels of vascular oxidative stress, inflammation, and endothelial dysfunction. In addition, the WHFDS rats displayed significantly reduced activity of Nrf2 at the vascular level. CN significantly reverted endothelial dysfunction in the aortas and the mesenteric arteries. In addition, CN significantly decreased vascular oxidative damage, inflammation at vascular and perivascular level and up-regulated Nrf2 activity in the arteries of WHFDS rats. Cinnamaldehyde, an activator of Nrf2, can be used to improve metabolic profile, and to revert endothelial dysfunction in obesity and metabolic syndrome.

## 1. Introduction

Obesity is an epidemic disease with a growing prevalence worldwide [1]. Metabolic syndrome (MetS) is a condition associated with at least three of the following cardiovascular risk factors: central adiposity; hypertension; reduced high-density lipoprotein cholesterol (cHDL) levels; increased triglyceride levels; and dysglycaemia [2]. Worldwide, the prevalence of MetS is rising (10–84%), and is interlinked with an increased risk of cardiovascular disease, type 2 diabetes, and non-alcoholic fatty liver disease [3]. MetS is increasingly recognized as a crucial public health problem. Insulin resistance, the low-grade pro-inflammatory state, the hypercoagulable/prothrombotic state, endothelial dysfunction, and the unbalanced redox state are also features of MetS [3,4].

NF-E2-Related Factor-2 (Nrf2), recognized as a crucial transcription factor in cellular protection against oxidative stress, is a master regulator of antioxidant and anti-inflammatory response [5,6]: controlling more than 250 genes, it confers protection against oxidative stress and inflammation [7]. Cinnamaldehyde (CN), an activator of Nrf2 [8], is a major compound found in the bark and leaves of some cinnamon varieties [9]. Cinnamon is commonly used as a spice in desserts, drinks, flavoring and traditional medicine [10,11,12,13]. Importantly, cinnamon has antioxidant [14], anti-inflammatory [15,16,17] and anti-diabetic [18] properties that are attributed to the cellular effects of CN. CN may have cardiovascular benefits, preventing reactive oxygen species (ROS) damage by up-regulation phase II detoxifying enzymes, and enhancing cellular glutathione [19,20]. Previous studies have revealed that CN has various pharmacological actions, including antihyperglycemic [21,22] and cardiovascular protective effects [23], suggesting that it may have a beneficial role in vascular disorders linked with metabolic syndrome. In a recent study, cinnamaldehyde exerted anti-atherosclerotic and anti-inflammatory effects in ApoE−/− mice [24].

The results of animal studies investigating the impact of Nrf2 modulation on obesity are controversial [25]. Recent studies suggest that CN may be atheroprotective in diabetic animal models; therefore, we hypothesized that CN could have beneficial effects on endothelial dysfunction associated with metabolic syndrome. The present study aimed to investigate the effects of CN on endothelial-dependent vasorelaxation in isolated rat aortas and the mesenteric arteries of Wistar rats fed with sucrose, a high-fat diet, or both. Metabolic profile, insulin resistance, vascular oxidative stress and inflammation at vascular and perivascular level were also evaluated. High-fat diets with or without sucrose induced endothelial dysfunction in normal Wistar rats, accompanied by the metabolic syndrome features. CN treatment normalized endothelial function, accompanied by a decrement in oxidative stress and vascular inflammation.

## 2. Methods

### 2.1. Animal Models

Wistar rats were originated from the local breeding colony (Faculty of Medicine, iCBR, University of Coimbra, Portugal). The rats were divided into 8 experimental groups: (1) W rats fed with a normal diet (W); (2) W rats fed with high-fat diet (WHFD); (3) W rats fed a 20% sucrose diet (WS); (4) WHFD fed with sucrose diet (WHFDS); (5) W treated with CN (WCN); (6) WS treated with CN (WSCN); (7) WHFD treated with CN (WHFDCN); and (8) WHFDS treated with CN (WHFDSCN). A special high-fat diet (HFD) was purchased from Safe (France), and contained 70% AO4 standard chow diet, 7.5% cocoa butter, and 1.25% cholesterol. The animals were kept on the HFD and/or a sucrose diet between 3 and 8 months of age. CN was administered intraperitoneally (20 mg/Kg) for 2 months (between 6- and 8-months-old) in all the groups. No animals older than 8 months were used.

The animal procedures and experiments were based on the principles of laboratory animal care, as adopted by the *EC Directive 86/609/EEC for* animal experiments.

### 2.2. Determination of Metabolic and Oxidative Stress Parameters

After an overnight fasten (around 15 h), the animals were anesthetized by a mixture of ketamine/chlorpromazine (75 mg/kg and 2.65 mg/Kg, respectively; im, Lab. Vitória, Portugal), followed by decapitation. Blood was collected by heart puncture. Fasting plasma lipids (total and HDL cholesterol and triglycerides, Olympus-Diagnóstica Portugal, Lisbon, Portugal) were quantified, using commercially available kits. Plasma free fatty acids (FFA) levels were determined, using enzymatic assay kits (Roche Applied Science, Lisbon, Portugal). Using metabolic cages, urine samples were collected during the course of 24 h. Urinary 8-hydroxy-2′-deoxyguanosine (8-OHdG) and plasma malondialdehyde levels (MDA) were evaluated as previously [26,27,28].

For glucose tolerance tests, the rats were intraperitoneal-injected with a glucose load (1.75 g.kg^−1^ body weight) after an overnight fast. Blood glucose was determined as previously [26,29,30]. Insulin resistance was determined by calculation of triglyceride–glucose (TyG) index = ln [fasting triglycerides (mg/dL) × fasting blood glucose (mg/dL)/2)], a screening method for insulin resistance used in both rats and humans [31,32]. The adiposity index was obtained by adding the weights of white adipose tissues/body weight × 100.

### 2.3. Isometric Tension Studies

Aortas and second-order mesenteric arteries were isolated and mounted as previously described [26,29]. Briefly, the arteries were excised, freed of connective tissue and mounted in an organ bath or in myograph chambers filled with modified Krebs–Henseleit buffer [oxygenated (95% O_2_, 5% CO_2_), 37 °C, pH 7.4, with the following composition: NaCl 119 mM; KCl 4.7 mM; CaCl_2_ 1.6 mM; MgSO_4_ 1.2 mM; NaHCO_3_ 25 mM; KH_2_PO_4_ 1.2 mM; glucose 11.0 mM). After 60 min of equilibrium, all vessels were preconstricted with 0.3 µM phenylephrine. Endothelium-dependent relaxation was determined as previously described [26,29,30].

### 2.4. Active Nrf2 Binding Assay

Nuclei were extracted from the aortas and mesenteric arteries using the Nuclear Extract Kit (Active Motif, Carlsbad, CA, USA), and were used for the determination of Nrf2-binding activity, using a TransAM Nrf2 kit (Active Motif, Carlsbad, CA, USA) according to the manufacturer’s instructions [22].

### 2.5. Real-Time Polymerase Chain Reaction

Total RNA isolation from arteries was performed using the TRI Reagent^®^ method (MRC Inc., Charleston, WV, USA) as previously described [33]. Gene expression of target genes was normalized to that of housekeeping gene Abt1, GAPDH and Hprt. Changes in mRNA expression were evaluated by conventional RT-PCR [33].

### 2.6. Assessment of Artery Immunofluorescence

Sections (6 μm) of arteries were washed (PBS) and fixed (ice-cold acetone, 10 min). The sections were then permeabilized [1% Triton X-100 in PBS, pH 7.4 (10 min)], and blocked (10% goat serum, 30 min). The arterial sections were incubated with primary antibodies (PBS/0.02% BSA, overnight at 4 °C), washed and subsequently incubated with secondary antibodies (1 h at room temperature). 

Immunostained artery sections were counterstained with 4′,6-diamidino-2-phenylindole, mounted, examined, photographed and quantified as described [26,29,30].

### 2.7. Statistical Analysis

Data was expressed as mean ± SEM (n = 12 rats per group), and were analyzed by standard computer programs (GraphPad Prism PC Software version 3.0, ANOVA). Significant differences were evaluated using either the *t*-test or ANOVA; *p* < 0.05 was considered significant. Dose–response curves were fitted and compared. as previously described [26,29,30].

## 3. Results

### 3.1. Metabolic Parameters

**Figure 1:**
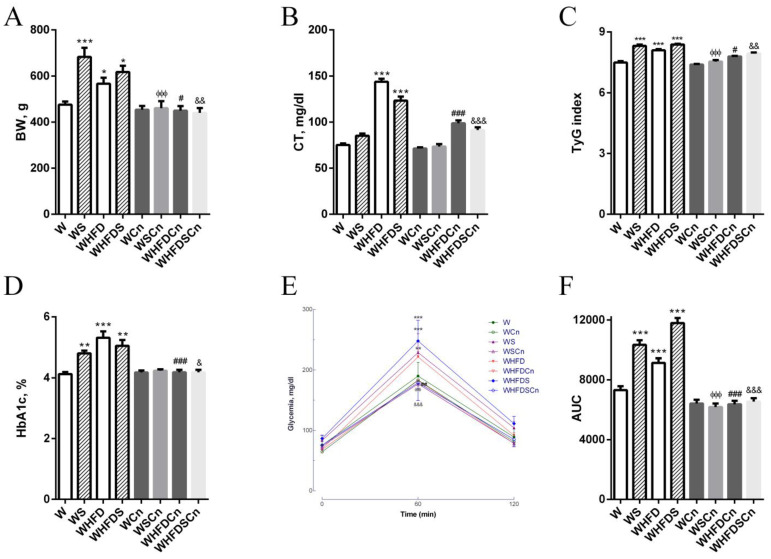
Effects of cinnamaldehyde treatment on: (**A**) body weight; (**B**) total cholesterol; (**C**) TyG index (an insulin resistance marker); (**D**) glycated hemoglobin levels; (**E**) intraperitoneal glucose tolerance test (IPGTT); and (**F**) the glucose area under the curve (AUC) in 8-months-old Wistar (W) rats fed with sucrose (WS), a high-fat diet (WHFD), or both (WHFDS), compared to normal W rats. (**E**). Data are expressed as mean ± SE (n = 12 animals per group). * *p* < 0.05, ** *p* < 0.01, *** *p* < 0.001 vs. W rats; ϕϕϕ *p* < 0.001 vs. WS rats; # *p* < 0.05, ### *p* < 0.001 vs. WHFD rats; & *p* < 0.05, && *p* < 0.01, &&& *p* < 0.001 vs. WHFDS rats.

Body weight, adiposity and TyG indexes were significantly higher in WS, WHFD and WHFDS rats compared to control W rats, and treatment with CN significantly reduced these parameters (Figure 1, Table 1). Following an intraperitoneal glucose tolerance test (IPGTT), WS, WHFD and WHFDS displayed noticeable glucose intolerance (Figure 1). HbA1c, the glucose area under the curve (AUC), triglycerides and free fatty acids were increased in the WS, WHFD and WFDS rats compared to the control W rats (Figure 1, Table 1). After 5 months of high-fat diet and sucrose feeding, the W rats exhibited significantly increased fasting blood glucose, glycated hemoglobin, plasma cholesterol, glucose intolerance and insulin resistance (Figure 1). Treatment with CN significantly improved the lipid profile (Figure 1, Table 1). Additionally, in MetS groups, treatment with CN for 8 weeks effectively decreased FFA levels in rats, and normalized fasting glucose (Table 1), glucose intolerance, HbA1c levels and the TyG index, an insulin resistance marker (Figure 1).

### 3.2. Vascular Function

**Figure 2:**
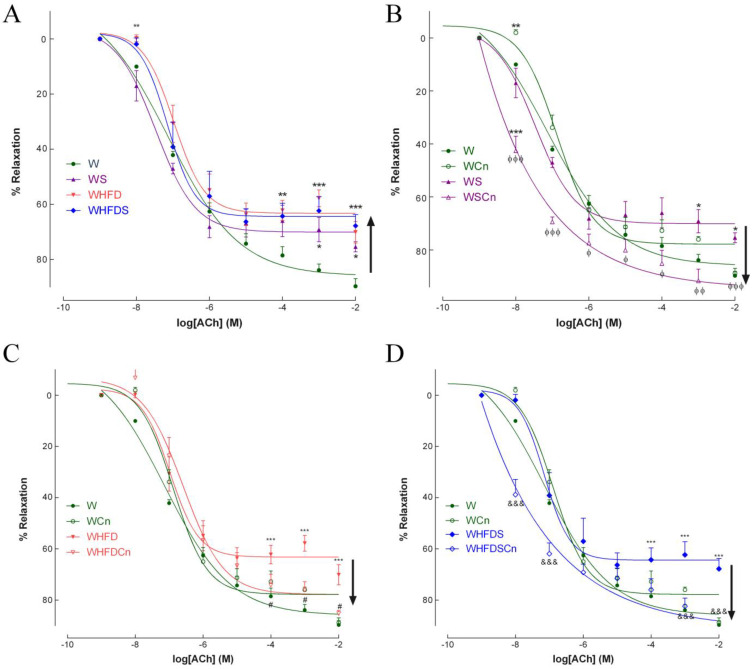
Effects of cinnamaldehyde (Cn) treatment on vasodilatory responses to acetylcholine in aortas of 8-months-old Wistar (W, **A**) rats fed with sucrose (WS, **B**), high-fat diet (WHFD, **C**) or both (WHFDS, **D**), compared to normal W rats. Data are expressed as mean ± SE (n = 12 animals per group). * *p* < 0.05, ** *p* < 0.01, *** *p* < 0.001 vs. W rats; ϕ *p* < 0.05, ϕϕ *p* < 0.01, ϕϕϕ *p* < 0.001 vs. WS rats; # *p* < 0.05 vs. WHFD rats; &&& *p* < 0.001 vs. WHFDS rats.

**Figure 3:**
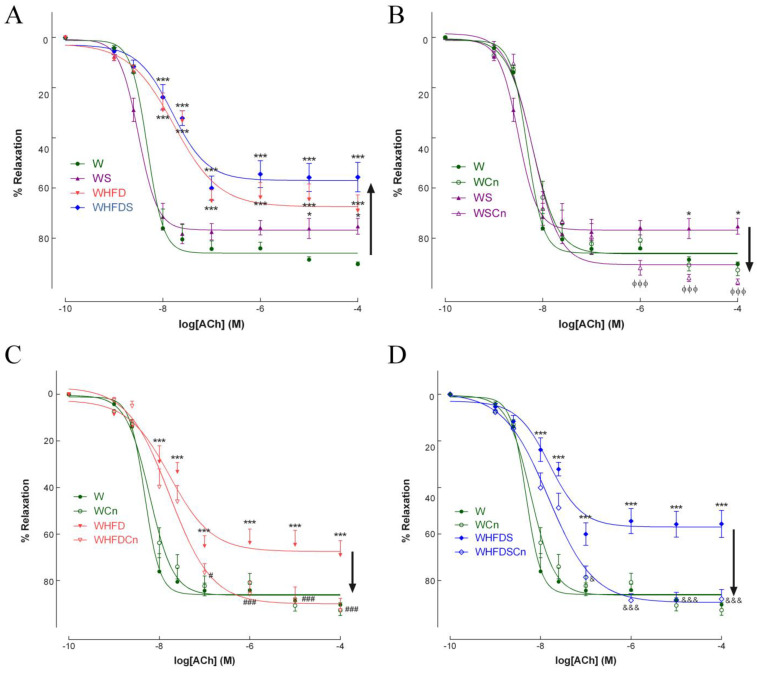
Effects of cinnamaldehyde (Cn) treatment on vasodilatory responses to acetylcholine in mesenteric arteries of 8-months-old Wistar (W, **A**) rats fed with sucrose (WS, **B**), high-fat diet (WHFD, **C**) or both (WHFDS, **D**), compared to normal W rats. Data are expressed as mean ± SE (n = 12 animals per group). * *p* < 0.05, *** *p* < 0.001 vs. W rats; ϕϕϕ *p* < 0.001 vs. WS rats; # *p* < 0.05, ### *p* < 0.001 vs. WHFD rats; & *p* < 0.05, &&& *p* < 0.001 vs. WHFDS rats.

In the 8-months-old WS, WHFD and WHFDS rats, endothelium-mediated vascular relaxation in response to ACh was impaired compared to the control rats (Figure 2A). Maximal endothelial-dependent relaxation of aorta rings in response to ACh declined by 25, 30 and 32% in the WS, WHFD and WHFDS rats, respectively (Figure 2A). N-nitro-L-arginine-methyl ester (L-NAME) and indomethacin dramatically reduced relaxation to ACh (Supplementary Figure S1A). Treatment with CN significantly recovered endothelium-dependent vascular relaxation in the aortas of all MetS groups (Figure 2B–D). The CN effect was abrogated after incubation with L-NAME plus indomethacin (Supplementary Figure S1A). In mesenteric arteries, maximal endothelial-dependent relaxation to ACh declined by 25, 34 and 44% in the WS, WHFD and WHFDS rats, respectively (Figure 3A). Treatment with CN reverted endothelial-dependent vasodilation in the mesenteric arteries of the WS, WHFD and WHFDS rats (Figure 3B–D). These data indicate that vascular endothelial dysfunction was induced by the direct effects of high-fat diet or high-sucrose diet, or both, in resistance and conduit vessels, and that CN treatment normalized endothelial function in these arteries (Figure 2 and Figure 3). Given the more pronounced phenotype of WHFDS regarding MetS features and vascular dysfunction, we focused on the mechanisms behind CN treatment in this group of MetS rats.

The specific role of endothelium-derived relaxing factors comprising NO, endothelium-derived hyperpolarization (EDH) or COX-derived prostanoids was determined (Supplementary Figure S1B,C) by concentration-response curves to acetylcholine after 30 min of incubation by their respective inhibitors: L-NAME (100 µmol/L); TRAM34 (T) plus apamin [(A)1 µmol/L each]; indomethacin (I, 10 µmol/L). In second-order mesenteric arteries, we observed that the contribution of NO to ACh-induced vasodilation was around 30%, EDH was around 40% and COX-derived prostanoids were approximately 16% (Supplementary Figure S1B). MetS rats exhibited endothelial dysfunction (the ACh-induced relaxation was 56% (22% NO dependent, 20% -EDH dependent and 14% COX-dependent); Supplementary Figure S1B). The mesenteric arteries of MetS rats treated with CN (WHFDSCn rats) normalized endothelial function. Endothelium-dependent relaxation in the WHFDSCn rats improved by 34% when compared to the MetS rats (WHFDS): in contrast, this response reduced to 20% in the presence of L-NAME, and was abolished in the presence of apamin plus TRAM34, indicating that CN affects both the NO and EDH components of endothelial-dependent relaxation (Supplementary Figure S1C).

### 3.3. Oxidative Stress Evaluation

**Figure 4:**
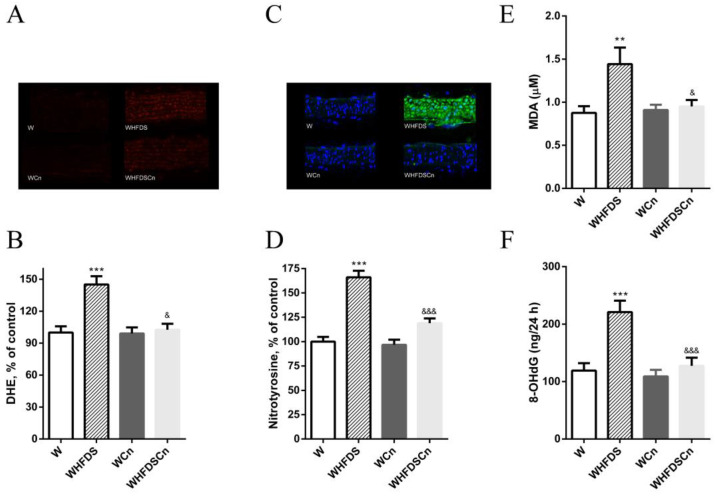
Effects of cinnamaldehyde (Cn) treatment on systemic and vascular oxidative stress in Wistar (W) rats fed with sucrose and high-fat diet (WHFDS) compared to normal W rats: (**A**) aorta artery sections representative of DHE-stained (O_2_^•−^ production) in different groups; the endothelium is facing up; at identical settings, WHFDS fluorescence was significantly increased, reflecting O_2_^• −^ levels in all layers of WHFDS aortas compared to normal W vessel; DHE fluorescence decreased in the WHFDS rats treated with Cn (WHFDSCn); (**B**) provides quantification of the red fluorescence; (**C**) mesenteric sections representative of nitrotyrosine staining in normal W, WHFDS treated with Cn (WHFDSCn) or without Cn; (**D**) provides quantification of the green fluorescence; (**E**) plasma malondialdehyde (MDA); and (**F**) urinary 8-hydroxydeoxyguanosine (8-OHdG) levels in the various groups of rats. Data are expressed as mean ± SE (n = 12 animals per group). ** *p* < 0.01, *** *p* < 0.001 vs. W rats; & *p* < 0.05, &&& *p* < 0.001 vs. WHFDS rats.

In metabolic syndrome rats, we determined the potential impact of CN on oxidative stress biomarkers. HFD and sucrose led to a 1.5-fold increase in superoxide levels in aortas (Figure 4A,B). Dihydroethidium (DHE) fluorescence was significantly reduced in the aortas of the WHFDS rats treated with CN, compared to the control rats (Figure 4A,B). Additionally, the WHFDS rats also exhibited higher immunoreactive nitrotyrosine levels in their mesenteric arteries (Figure 4C,D), and CN treatment significantly decreased these levels (Figure 4C,D). CN decreased vascular oxidative injury in metabolic syndrome rats in both arteries.

Plasma MDA (a biomarker of lipid peroxidation) and urinary levels of 8-OHdG (an indicator of DNA damage) were significantly increased in the WHFDS rats, when compared to the control rats (Figure 4E,F). Treatment with CN for 8 weeks significantly reduced MDA and 8-OHdG levels (Figure 4E,F).

### 3.4. eNOS Expression Levels

The eNOS expression levels did not significantly change in the aortas or mesenteric arteries of the MetS rats when compared to the W rats (Supplementary Figure S2A,B). The eNOS expression levels were significantly increased in both arteries after treatment with CN (Supplementary Figure S2A,B). Stimulation of aortas by ACh (10 mM) increased the levels of NO metabolites (Supplementary Figure S2C, right bar). Unstimulated values (Supplementary Figure S2C, left bars) did not change. NO metabolites in the aortic tissue of the MetS rats were significantly decreased relative to the W control values, and CN treatment restored these values (Supplementary Figure S2C). In addition, supplementation with CN completely restored NO metabolites in the serum of the WHFDSCn rats (Supplementary Figure S2D).

### 3.5. Inflammation in Aorta and Mesenteric Arteries and in PVAT

**Figure 5:**
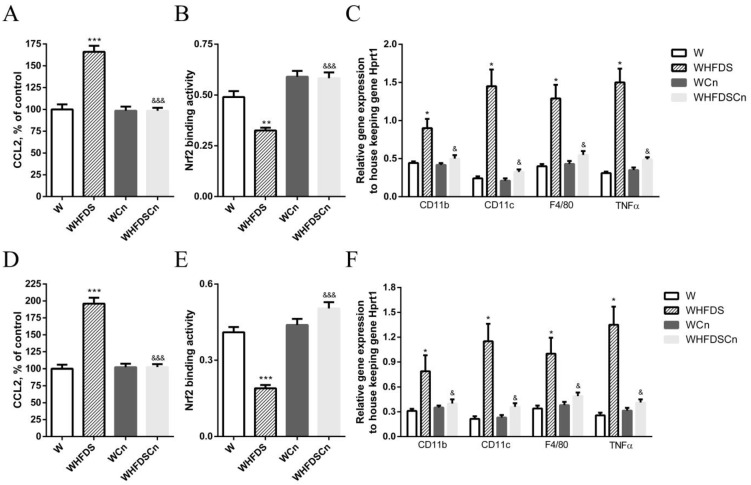
Effects of cinnamaldehyde (Cn) treatment on inflammatory markers and Nrf2 activity in the aortas (**A**–**C**) and mesenteric arteries (**D**–**F**) of Wistar (W) rats fed sucrose and high-fat diet (WHFDS), compared to normal W rats. Chemokine (C-C motif) ligand 2 (CCL2) levels (**A**,**D**), Nrf2 activity (**B**,**E**) and mRNA expression levels (**C**,**F**) of indicated genes in PVAT (**E**,**F**) of thoracic aortas and mesenteric arteries of WHFDS rats compared with normal W rats. Data are expressed as mean ± SE (n = 12 animals per group). * *p* < 0.05, ** *p* < 0.01, *** *p* < 0.001 vs. W rats; & *p* < 0.05, &&& *p* < 0.001 vs. WHFDS rats.

We have previously shown that mediators of inflammation are increased in arteries of type 2 diabetic rats, contributing to vascular dysfunction [26,29,30]. In this study, levels of the chemokine (C-C motif) ligand 2 (CCL2), an initial biomarker of inflammation in atherogenesis, were significantly higher in the aortas and mesenteric arteries of WHFDS rats compared to age-matched controls (Figure 5A,D). In addition, this pro-inflammatory marker was significantly decreased in both arteries of CN-treated WHFDS rats (Figure 5A,D).

Gene expression of pro-inflammatory cytokines was determined in the perivascular adipose tissue (PVAT) using quantitative polymerase chain reaction (PCR). Classically, M1 macrophages express CD11c, and predominate in the epidydimal adipose tissue of obese animals producing high levels of proinflammatory various cytokines such as TNFα [34]. PVAT analysis showed elevated levels of the proinflammatory genes CD11c, CD11b, F4/80 and TNFα in the aortic and mesenteric PVAT of the WHFDS rats relative to the control rats. CN significantly reduced the expression levels of these proinflammatory genes (Figure 5C,F).

CN restored Nrf2 activity in the aorta and mesenteric arteries of the metabolic syndrome rats.

A decline in Nrf2 function has been linked with metabolic syndrome, but it is not clear if CN can restore Nrf2 activity in WHFDS rats. We have previously shown that Nrf2 activity is enhanced after sulforaphane treatment in arteries from type 2 diabetic Goto-kakizaki rats [22]; therefore, we compared Nrf2 ARE-binding activity in the aorta and mesenteric arteries of W control and WHFDS rats: in both arteries, Nrf2 activity was significantly decreased in the WHFDS rats compared to the controls, and treatment with CN upregulated Nrf2 activity (Figure 5B,E).

## 4. Discussion

In the current study, we investigated the effects of CN and its underlying mechanisms on vascular dysfunction in metabolic syndrome rats. The therapeutic potential of cinnamaldehyde was clearly demonstrated in the metabolic syndrome endothelial dysfunction of diet-induced obesity in rats fed with HFD and/or sucrose. Treatment of WS, WHFD, and WHFDS rats with CN significantly improved endothelial dysfunction in the aortas and mesenteric arteries. CN treatment reverted the pathological characteristics of metabolic syndrome and endothelial dysfunction. CN was able to reduce vascular oxidative damage and inflammatory biomarkers in WHFDS, explaining the normalization of endothelial function achieved under metabolic syndrome conditions by the activation of the Nrf2 pathway. Importantly, we demonstrated for the first time that CN reduces PVAT inflammatory profile in the arteries of metabolic syndrome models.

W rats fed a high-fat diet and/or high sucrose are frequently used animal models of metabolic syndrome. Chronic exposure to western-type diets in these animals induces characteristics that resemble features seen in humans. The WHFDS model shares several cardiovascular phenotypes with human metabolic syndrome, including abnormal vascular reactivity, defending its use as the main model for this study.

Herein, we show that WHFDS rats are glucose intolerant and insulin resistant, with significantly increased body weight, adiposity index, HbA1c, total cholesterol and circulating levels of FFA. Endothelial dysfunction is present in the resistance and conduit arteries associated with increased oxidative stress, inflammation at vascular and perivascular level and depletion of Nrf2 levels.

Nrf2 antioxidant functions may be crucial in vascular disorders [22,35,36]. CN, an activator of Nrf2 found in cinnamon, exerted anti-atherosclerotic and anti-inflammatory effects in ApoE−/− mice [24]. The concentration of CN used was decided based on previous reports [37], and on its bioavailability in cinnamon [38]. Indeed, CN could be therapeutically attractive for the management of endothelial dysfunction at physiological doses that can be found in the human diet [39].

The field of MetS and its vascular complications requires novel and more suitable drugs to prevent cardiovascular disorders. CN has great potential in this field. Treatment with CN for 8 weeks significantly reduced body weight, adiposity index and triglyceride, total cholesterol and FFA levels. CN corrected hyperlipidemia and adiposity in both WHFD and WHFDS rats. This lipid-lowering effect was reported for higher concentrations of CN [21,40] and in other models of chronic disease [13,41]. Indeed, it was previously reported that CN significantly decreased triglycerides and total cholesterol, and increased in high-density lipoprotein-cholesterol in both STZ-induced diabetic rats, db/db transgenic mice [42] and patients with diabetes [10]. Using structure–activity relationship studies (α-β-unsaturated aldehyde functional group in CN binds covalently with thiol groups of Keap1, releasing and stabilizing Nrf2), the hypoglycemic and hypolipidemic effects of CN were previously proved [43]. Nrf2 activation decreases plasma glucose levels in wild-type but not in Nrf2-deficient mice [44]. Accordingly, CN treatment significantly improved glucose intolerance. Targeting Nrf2 with CN effectively reduced blood glucose and HbA1c levels, consistent with reports from other studies [42]. Indeed, CN has been described to promote cellular uptake of glucose and glycogen biosynthesis, and to improve insulin resistance and dysfunction of pancreatic islets [45,46,47,48,49].

Accordingly, our study demonstrates that CN treatment significantly reduces the insulin resistance index TyGR. Previous studies have shown that CN up-regulates the expression of insulin receptor genes [50], and increases the expression levels of the peroxisome proliferator-activated receptor-γ- activating AMP kinase, and consequently leads to an increment in insulin sensitivity [51,52].

The role of oxidative stress in the etiology of endothelial dysfunction is well-established [53]. Cardiovascular risk factors increase ROS in the vasculature, compromise the antioxidant defense enzymes, and attenuate the levels of intracellular antioxidants [19], promoting an increment in oxidative stress [53,54,55]. Previous studies have shown that activators of Nrf2 re-establish redox homeostasis, by enhancing the antioxidant/electrophilic-response-element-mediated expression of phase II and antioxidant enzymes [56,57]. In this study, we present evidence that treatment with CN exerts anti-oxidative effects in vivo, namely through the reduction of plasma MDA levels and urinary 8-OHdG, along with a decrement in tissue O_2_^•−^ anion and nitrotyrosine accumulation in metabolic syndrome rats. This anti-oxidative effect is crucial to explaining the recovery of endothelial function. Other studies have described the protective effects of CN on oxidative stress under metabolic syndrome [58,59,60]. In addition, it was previously reported that CN protects against diabetic vascular dysfunction by inhibiting oxidative stress through the activation of Nrf2 signaling in diabetic db/db mice [24]. Herein, we show that oxidative stress in MetS rats reduced NO metabolites in the serum of MetS rats, without changing eNOS expression levels in aortas and mesenteric arteries. CN treatment was able to reduce oxidative stress and, in addition, increased the expression levels of eNOS. Moreover, CN was also able to improve endothelial function in the presence of L-NAME, in mesenteric arteries, highlighting other mechanisms involved. Oxidative stress was increased in arteries, which was likely due to Nrf2-dependent reduction in antioxidant capacity. We proved, in vivo, that treatment with CN normalizes vascular function in both aortas and mesenteric arteries.

Moreover, CN significantly reduced vascular oxidative stress and inflammation, partially explaining the improvement in vascular function. CN is highly effective in decrementing inflammation at vascular and perivascular level. Our findings show that prolonged exposure to CN attenuated increased levels of nitrotyrosine and CCL2, biomarkers of inflammation in the arteries of metabolic syndrome rats. Additionally, we also observed that CN treatment alleviated the elevation of inflammation in the PVAT of aortas and in the mesenteric arteries of WHFDS rats. Consistently, it was previously found that CN attenuated ROS production and IL-1β secretion in macrophages [61,62]. In addition, CN enhanced the suppression of TNFα–induced monocyte/endothelial cell interactions [15,63].

The effects of CN on Nrf2-dependent gene expression are known [24,64]. WHFDS rats show a reduction in Nrf2 at the vascular level. Accordingly, decreased levels of Nrf2 have been described in animal models and human tissues [65,66,67]. In addition, aging is accompanied by Nrf2 dysfunction in the vasculature, increasing oxidative stress injury [68]. Accordingly, we also found that Nrf2 activity in arteries was significantly reduced in metabolic syndrome vasculature probably, leading to a decrement in antioxidant defense mechanisms [36].

Exploiting Nrf2 activators and their potential benefits may uncover a novel therapeutic strategy for restoring vascular function in metabolic syndrome.

## 5. Conclusions

In summary, we have demonstrated for the first time that CN was able to revert high-fat/high-sucrose-diet-induced endothelial-dependent vasorelaxation impairment, by a mechanism involving the activation of Nrf2. The pleiotropic effects of CN include antioxidant functions reducing oxidative stress and anti-inflammatory properties in the vascular wall and surrounding perivascular adipose tissue.

The therapeutic potential of CN to recover vascular damage induced by western-type diets is evident. Decreasing inflammation is simultaneous with the activation of Nrf2-restored vascular function in metabolic syndrome rats. Chronic dietary CN may represent a promising intervention in metabolic syndrome.

## Figures and Tables

**Table 1:** Effect of cinnamaldehyde treatment on adiposity index, fasting blood glucose, triglycerides and free fatty acid levels in W control rats, W fed with sucrose (WS), high-fat diet (WHFD), or both (WHFDS).

	W	WS	WHFD	WHFDS	WCn	WSCn	WHFDCn	WHFDSCn
**Adiposity index (%)**	14.15 ± 1.29	20.8 ± 1.05 *	27.3 ± 1.65 ***	32.6 ± 1.89 ***	10.1 ± 0.4	9.1 ± 1.1 ϕϕϕ	11.9 ± 0.9 ###	12.6 ± 1.2 &&&
**Fasting glucose (mg/dl)**	75.0 ± 0.81	83.25± 2.99 *	75.49 ± 1.01	86.5 ± 1.76 **	64.33 ± 0.97	68 ± 2.17 ϕϕϕ	68.39 ± 1.01	75.44 ± 2.15 &
**Triglycerides (mg/dL)**	48.75 ± 3.22	98.88 ± 1.16 ***	97.47 ± 4.7 ***	98..2 ± 4.17 ***	50.56 ± 1.86	56.22 ± 1.53 ϕϕϕ	65.89 ± 1.54 ###	75.22 ± 0.99 &
**Free fatty acids (mM)**	0.6 ± 0.02	1.2 ± 0.06 **	1.8 ± 0.15 ***	2.1 ± 0.11 ***	0.7 ± 0.13	0.59 ± 0.14 ϕ	0.7 ± 0.12 ###	0.76 ± 0.11 &&&

Adiposity index = sum of weights of white adipose tissues divided by body weight × 100. Data are expressed as mean ± SE (n = 12 animals in each group). * *p* < 0.05, ** *p* < 0.01, *** *p* < 0.001 vs W rats; ϕ *p* < 0.05, ϕϕϕ *p* < 0.001 vs WS rats; ### *p* < 0.001 vs WHFD rats; & *p* < 0.05, &&& *p* < 0.001 vs WHFDS rats.

## Data Availability

Data is contained within the article and the supplementary.

## Supplementary Materials

The following supporting information can be downloaded at: https://www.mdpi.com/article/10.3390/antiox12010082/s1. Figure S1. Effects of cinnamaldehyde (Cn) treatment on vasodilatory responses to acetylcholine in aorta (A) and mesenteric arteries (B,C) of 8 months old Wistar (W) rats fed with sucrose and high-fat diet (WHFDS) compared with normal W rats. The specific role of endothelium-derived relaxing factors including NO, EDH, or COX-derived prostanoids was evaluated by performing concentration-response curves to acetylcholine after 30 min of chamber incubation with their respective inhibitors: L-NAME (100 μmol/L), TRAM34 (T) plus apamin [(A)1 μmol/L each], indomethacin (I, 10 μmol/L). Data are expressed as mean ± SE (n = 12 animals in each group). & *p* < 0.05, &&& *p* < 0.001 vs WHFDS rats; $ *p* < 0.05, vs W+L-NAME; ΔΔ *p* < 0.01, ΔΔΔ *p* < 0.001 vs WHFDS+L-NAME. Figure S2. Effects of cinnamaldehyde (CN) on endothelial nitric oxide synthase (eNOS) expression levels in aorta (A) and mesenteric arteries (B). (C) NO metabolites were assessed in aortic homogenates and serum (D) using the Griess reaction. In each group of aortic homogenates, left and right bars represent basal and acetylcholine (ACh)-stimulated NO synthesis, respectively. Data are expressed as mean ± SE (n = 12 animals in each group). * *p* < 0.05, ** *p* < 0.01 vs W rats; & *p* < 0.05 vs WHFDS rats.
