# Supplementary material for: Cinnamaldehyde Supplementation Reverts Endothelial Dysfunction in Rat Models of Diet-Induced Obesity: Role of NF-E2-Related Factor-2
Source: Antioxidants (Basel). 2022 Dec 30;12(1):82. doi: 10.3390/antiox12010082 (PMC9854673; doi:10.3390/antiox12010082)
Supplement: Supplementary file 1 [file antioxidants-12-00082-s001.zip › antioxidants-2102406-supplementary.pdf]

Cinnamaldehyde Supplementation Reverts Endothelial Dysfunction in Rat Models of Diet-Induced Obesity: Role of NF-E2-Related Factor-2

Cristina M. Sena[1], Ana Pereira[1] and Raquel M. Seiça[1]

[1] Institute of Physiology, iCBR, Faculty of Medicine, University of Coimbra, Sub-unidade 1, Pólo III, Azinhaga de Santa Comba, Celas, 3000-548 Coimbra, Portugal

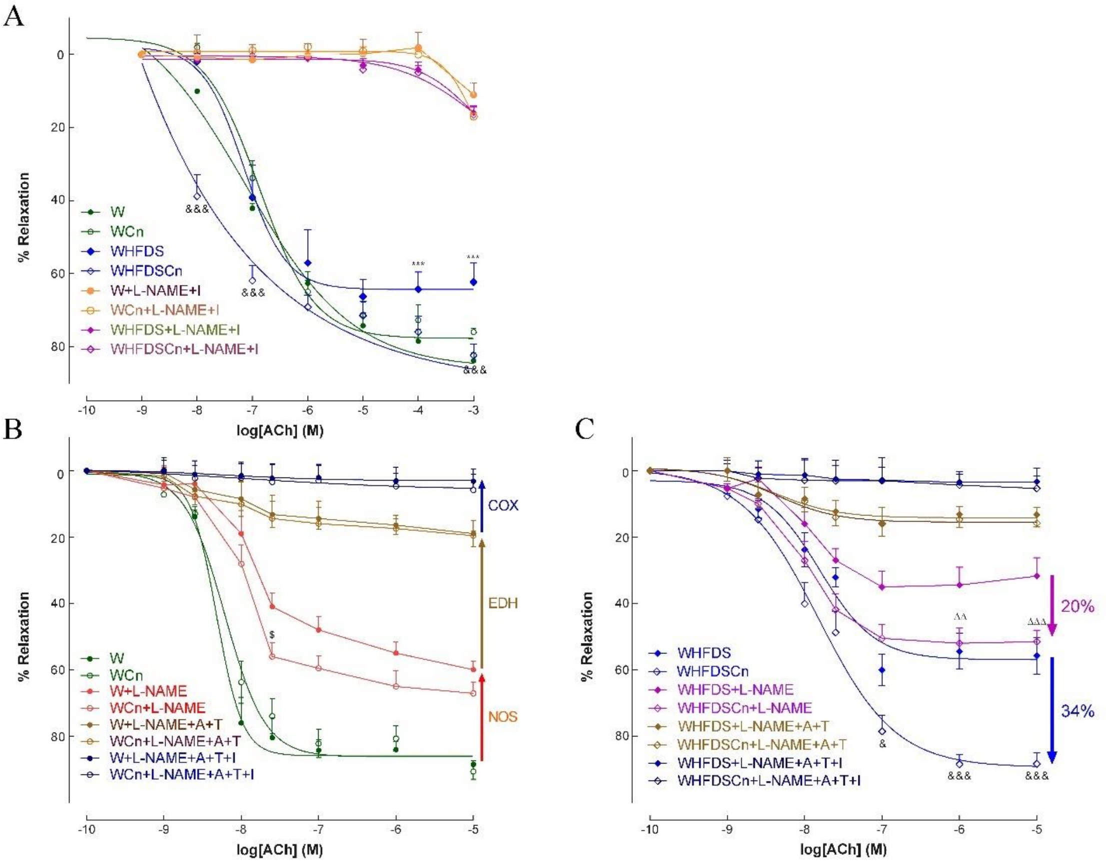

Supplementary Figure S1. Effects of cinnamaldehyde (Cn) treatment on vasodilatory responses to acetylcholine in aorta (**A**) and mesenteric arteries (**B,C**) of 8 months old Wistar (W) rats fed with sucrose and high-fat diet (WHFDS) compared with normal W rats. The specific role of endothelium-derived relaxing factors including NO, EDH, or COX-derived prostanoids was evaluated by performing concentration-response curves to acetylcholine after 30 minutes of chamber incubation with their respective inhibitors: L-NAME (100 μmol/L), TRAM34 (T) plus apamin [(A)1 μmol/L each], indomethacin (I, 10 μmol/L). Data are expressed as mean ± SE (n = 12 animals in each group). &P<0.05, &&&P<0.001 vs WHFDS rats; $P<0.05, vs W+L-NAME; ΔΔP<0.01, ΔΔΔP<0.001 vs WHFDS+L-NAME.

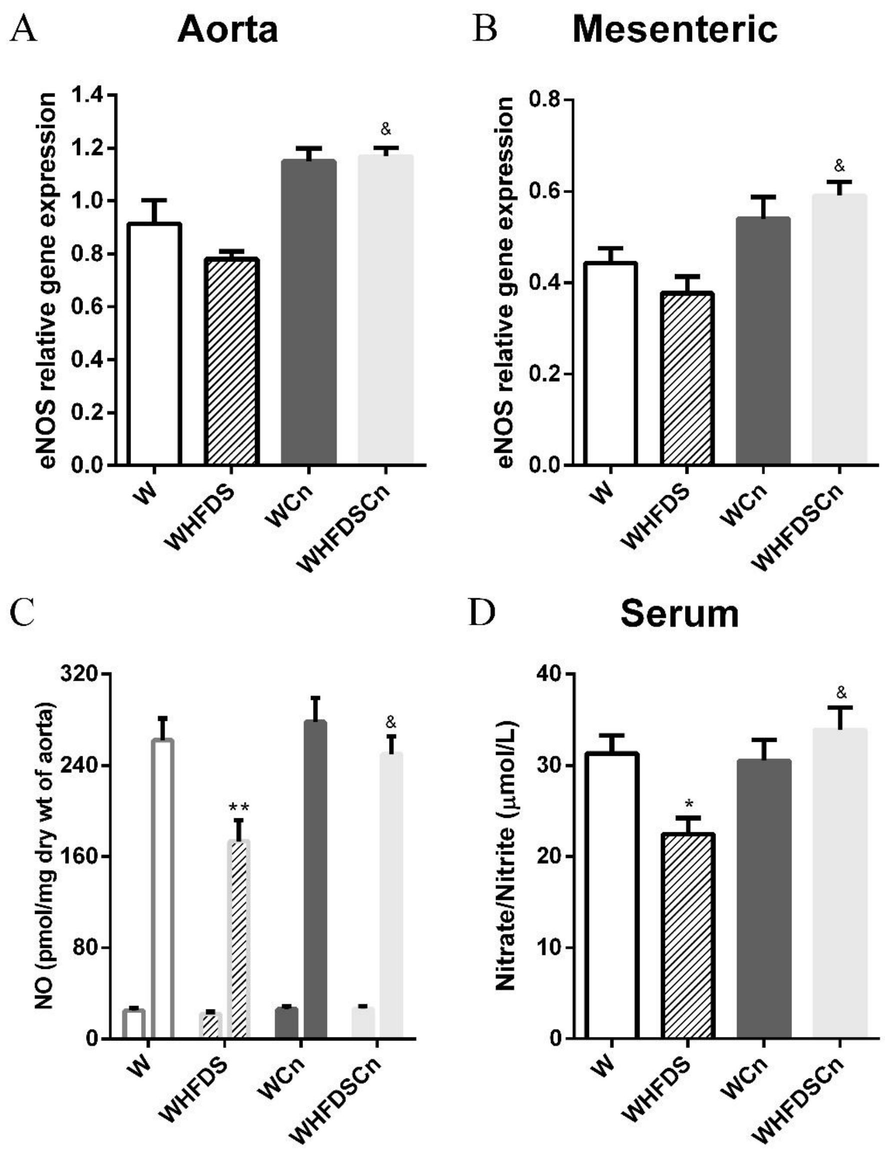

Supplementary Figure S2. Effects of cinnamaldehyde (CN) on endothelial nitric oxide synthase (eNOS) expression levels in aorta (**A**) and mesenteric arteries (**B**). (**C**) NO metabolites were assessed in aortic homogenates and serum (**D**) using the Griess reaction. In each group of aortic homogenates, left and right bars represent basal and acetylcholine (ACh)-stimulated NO synthesis, respectively. Data are expressed as mean ± SE (n = 12 animals in each group). *P<0.05, **P<0.01 vs W rats; &P<0.05 vs WHFDS rats.
